# Pacopampa: Early evidence of violence at a ceremonial site in the northern Peruvian highlands

**DOI:** 10.1371/journal.pone.0185421

**Published:** 2017-09-28

**Authors:** Tomohito Nagaoka, Kazuhiro Uzawa, Yuji Seki, Daniel Morales Chocano

**Affiliations:** 1 Department of Anatomy, St. Marianna University School of Medicine, Kawasaki, Japan; 2 Faculty of Human Sciences, University of East Asia, Shimonoseki, Japan; 3 National Museum of Ethnology, Suita, Japan; 4 Universidad Nacional Mayor de San Marcos, Lima, Peru; Seoul National University College of Medicine, REPUBLIC OF KOREA

## Abstract

**Objectives:**

Pacopampa, a ceremonial complex in Peru’s northern highlands, reveals early evidence of trauma in the Middle to Late Formative Period coinciding with the emergence of social stratification in the area. We examine the prevalence of trauma in human remains found at the site and present evidence of the circumstances surrounding the deaths of individuals who lived during the early stages of Andean civilization.

**Materials and methods:**

The materials are the remains of 104 individuals (38 non-adult and 66 adult) from the Middle to Late Formative Periods. We explored trauma macroscopically and recorded patterns based on skeletons’ locations, age at death, sex, social class, and chronology.

**Results:**

We detected trauma in remains over the Middle to Late Formative Periods. While the prevalence of trauma was minimal in the Middle Formative Period, skeletons from the subsequent era exhibit more severe disturbances. However, all the skeletons show signs of healing and affected individuals experienced a low degree of trauma.

**Discussion:**

Given the archaeological context (the remains were recovered from sites of ceremonial practices), as well as the equal distribution of trauma among both sexes and a lack of defensive architecture, it is plausible that rituals, rather than organized warfare or raids, caused most of the exhibited trauma. Pacopampa was home to a complex society founded on ritual activity in a ceremonial center: this is indicated by the presence of ritual violence in a society that built impressively large, ceremonial architecture and developed social stratification without any political control of surplus agricultural goods.

## Introduction

Mortality and morbidity caused by trauma are clues to assessing how various cultures and societies may have influenced behavior in their members [1]. Although archaeological remains of fortifications, defensive architecture, settlement patterns, weapons, iconography, and symbols hint at past violent behavior, human skeletons with traumatic injuries (e.g., spear injuries, decapitations, mutilations, scalping, and depressed skull fractures) serve as direct evidence [1].

Andean civilization experienced multiple empires until the end of Inca rule in 1532, and was closely linked with violence throughout, starting in the Archaic Period (8000–1800 BC) [2]. People in the Archaic Period relied on a mixed subsistence system that, while it varied across time and space, included: gathering, hunting, and fishing; cultivating cotton, potato, manioc, sweet potato, ullucu, jicama, oca, capsicum, lúcuma, lima beans, quinoa, cucurbita, lagenaria, coca, peanuts, and maize [3]; and domesticating camelids and guinea pigs [4]. The first proof of trauma possibly caused by violence in the Andes region was found among human remains from Chinchorro (7000–1600 BC) in northern Chile, in the South Andes [5], where most of the skeletons’ injuries were concentrated in the crania, and 24.6% of the adults exhibited healed skull fractures [5]. Cranial trauma was three times more prevalent in males than females; clustering on the left side of the face and cranial vaults indicating interpersonal fighting [5]. It is possible that fights in Chinchorro resulted from competition over natural resources or comprised mock fighting as part of funeral rites, rather than accidents [5]. In addition, the oldest evidence of human sacrifice in the region, dating back to 5000 BC, appeared on the central coast of Peru: a number of children were buried under houses as a ritual infanticide practice at the site of La Paloma [6].

To evaluate the traces of violence observed on human skeletons, the socio-economic background of prehistoric societies has been explored through archaeological evidence, including architectural remains, settlement patterns, and subsistence studies. The first construction of public architecture in the Central Andes has been dated to about 3000 BC, which some scholars define as the beginning of the Formative Period [7]. This period involved cultural changes, including the advent of sedentism and social complexity [7]. Both direct and indirect evidence of violence in the Central Andes was first found in this period, but such findings have, to date, been very rare. The large monumental complexes that combine platform mounds and associated sunken circular plaza were constructed in the Supe Valley, on the central coast of Peru [8]. Excavations at Caral, one of the complexes in Supe, yielded an isolated cranium; it was inferred that the remains belonged to a sacrificial victim [9], but analysis of the remains did not include a bioarchaeological description of the trauma. Also, the bodies of infants were buried with beads around their heads at the site of Aspero in Supe on the central coast of Peru [10]. Engel [11] excavated two headless adults and seven isolated non-adult and adult crania from Asia Beach (1300–1100 BC), on the central coast of Peru. These remains included decapitated crania with cut marks on the cranial vault and face, considered to be attributable to sacrifice [11]. At the site of Cerro Sechín (1500 BC), on the northern coast of Peru, a stone carving bears a drawing depicting violence and organized warfare [12]; however, there are no direct signs of violence on the northern coast of Peru until the Final Formative Period [2].

In the Middle to Late Formative Periods (1200–500 BC), an impressive array of various kinds of large ceremonial architecture was built, despite the lack of an intensive farming system (for instance, involving large-scale irrigation and storage installations for surplus agricultural goods), at least in the northern highlands of Peru. The background of social development in the Central Andes is characterized by the construction and renovation of ceremonial architecture, which had been continuously rebuilt via collaborative efforts, without any political control of surplus agricultural products [13]. Buried skeletons found at the ruins of Kuntur Wasi [14] and Pacopampa [15] in the northern highlands showed indications of being linked to great wealth, signaling that they belonged to an elite class and that stratified societies existed there. However, evidence of violence before 500 BC has rarely been detected [2]. No cranial trauma has been found in the Bolivian highlands [16] or along Peru’s northern coast [17]. There are a few cases of ritual offerings of human skull in the sites at Cardal [18] on the central coast of Peru and at Kotosh [19] and Shillacoto [20] in the Peruvian central highlands, but analyses of the human remains did not include bioarchaeological descriptions of the trauma. Possible direct proof of violence was discovered among five female crania from the site of Wichqana in the southern highlands of Peru (1150–750 BC): these remains exhibited artificial cranial deformations along with trauma [21]. An association of these crania with mandibles and cervical vertebrae suggests perimortem decapitation, but it is unknown whether they were victims of sacrifice [21]. In addition, a male cranium from Kutur Wasi (800–550 BC), which was buried with copper and bone artifacts, exhibited a lethal blow to the left temporal bone with an blunt object [14,22]. However, the settlement patterns of the Middle to Late Formative Periods lacked defensive architecture, implying that the inhabitants led generally peaceful lives [2]. Nevertheless, contemporaneous iconographic motifs favor figures of warriors, weapons, and trophy heads [23]. Trophy heads were drawn abstractly on ceramics found at ceremonial monuments at the ruins of Huacaloma [24] and Pacopampa [25]. Cross-legged supernatural beings holding trophy heads were also portrayed on a monolith recovered at Kuntur Wasi [14].

During the Final Formative Period (500–50 BC), the domestication of camelids increased radically at Huacaloma in the Cajamarca Basin [26]. Stable isotope data from human remains from various sites in Peru were found to indicate less consumption of maize in the Formative Period than expected. Seki and Yoneda [27] analyzed human skeletons from the Formative Period sites of Kuntur Wasi, Loma Redonda, Huacaloma, and Kolguitín in the northern highlands. Discussing maize consumption in Formative Period societies based on carbon and nitrogen stable isotope analysis, they concluded that although those societies grew maize, their reliance on it was not notable. These data imply an increase in maize consumption during the Formative Period, but the intensification of its cultivation did not occur until subsequent eras.

Although there is no direct evidence for violence in the northern highlands, an anecdotal description of trophy heads drawn on ceramics found at the ruins of Nasca revealed that conflict did exist on the southern coast of Peru [2]; in addition, actual trophy heads were recovered from the Palpa Valley and the Acari Valley [28, 29]. These headless individuals showed signs of projectile injuries and did not have goods in their graves, suggesting that the trophy heads were taken from enemies during conflict [28, 29]. These findings are consistent with the increase in defensive settlements and cranial trauma that occurred during the Final Formative Period [2].

The abovementioned studies show that there may have been trauma related to violence, at least during the Formative Period, along the coast in the Central Andes. Violence-related trauma in the highlands first appeared during the Middle to Late Formative Periods, later than on the coast. However, the timing of the emergence of violence in a ritual context remains unknown, as do the social changes that accompanied it during the nascent stage of complex societies in this region. This is because most studies have not provided direct proof of violence and the archaeological contexts of recovered human skeletons have not coincided with the beginning of social complexity. The ceremonial complex at Pacopampa provides the oldest evidence of trauma in the northern highlands, which coincided with the emergence of social stratification in the area. Using the data with known archaeological contexts, we tested the hypothesis that death by violence represents the circumstances of the time and place, as well as the theory that trauma was caused by ritual activity, thus revealing violence in the early stages of Andean civilization.

### Archaeological settings at Pacopampa

In the 1960s, excavations from the Formative Period at the central highlands site of Kotosh demonstrated that the construction of ceremonial architecture preceded the manufacturing of pottery and influenced socioeconomic development [19]. Archaeological works on ceremonial architecture at Huacaloma in the northern highlands have verified that renovation activities were important for social integration during the Formative Period [24]; however, social development based on the construction and renovation of ceremonial architecture did have limitations, as there is no evidence for social hierarchy at Huacaloma and other sites. This contrasts with evidence from recent investigations at Kuntur Wasi, which indicate that social differentiation existed there and was based on the long-distance trade of precious goods [13]. More than seven tombs have been unearthed at Kuntur Wasi and found to contain gold objects, Ecuadorian shell ornaments, and Bolivian sodalite beads. Moreover, these tombs have a complex, boot-shaped structure, and some of the individuals buried there show the cranial deformation and red pigment, which suggests that the individuals buried there had special social roles as members of an elite group.

**Fig 1:**
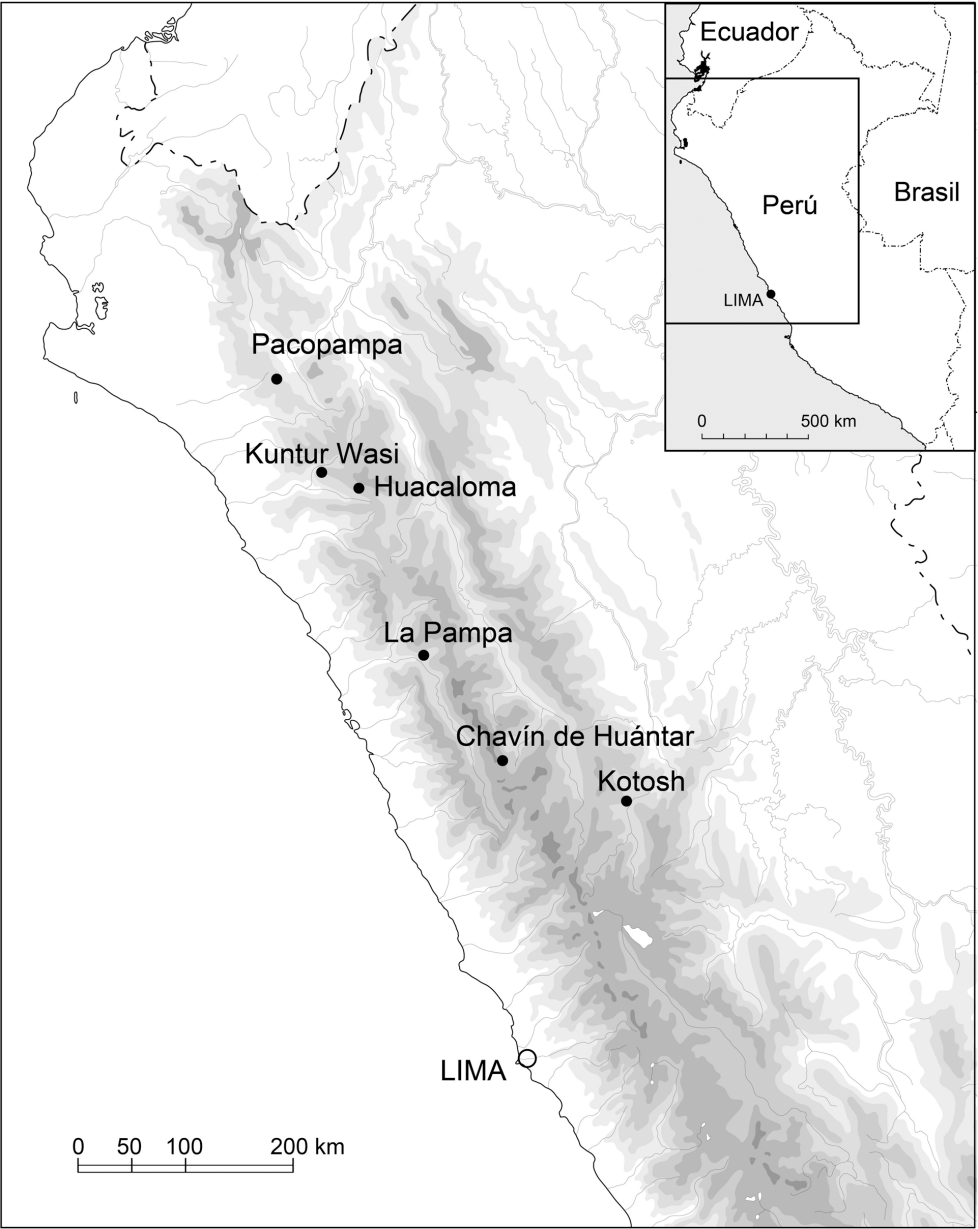
Map of Peru showing the location of Pacopampa.

Pacopampa is a Formative Period site in Peru’s northern highlands, at an altitude of 2,500 m above sea level (Fig 1). It is roughly 70 km from the Pacific coast and also distant from tropical riverbanks. Rafael Larco Hoyle, a Peruvian archaeologist, conducted the first excavation there in 1939. Further excavations occurred from the mid-20th century, yielding ceremonial architecture and cultural artifacts related to ritual practices. A small amount of marine shellfish was recovered, even though it is unlikely that the people who lived there ate them, since the site is far from the Pacific coast [30]. The elaborate subterranean canal systems seem to have played an important role in rituals related to water and agricultural production [30]. In addition, exotic pottery was found, including styles from the Pacific coast and the eastern slopes of the Andes Mountains [30]. This variety of foreign artifacts allows us to trace the wide-ranging connections kept by the ceremonial center and the patterns of exchange that were essential to maintaining the prestige of Pacopampa and its elites [30].

**Fig 2:**
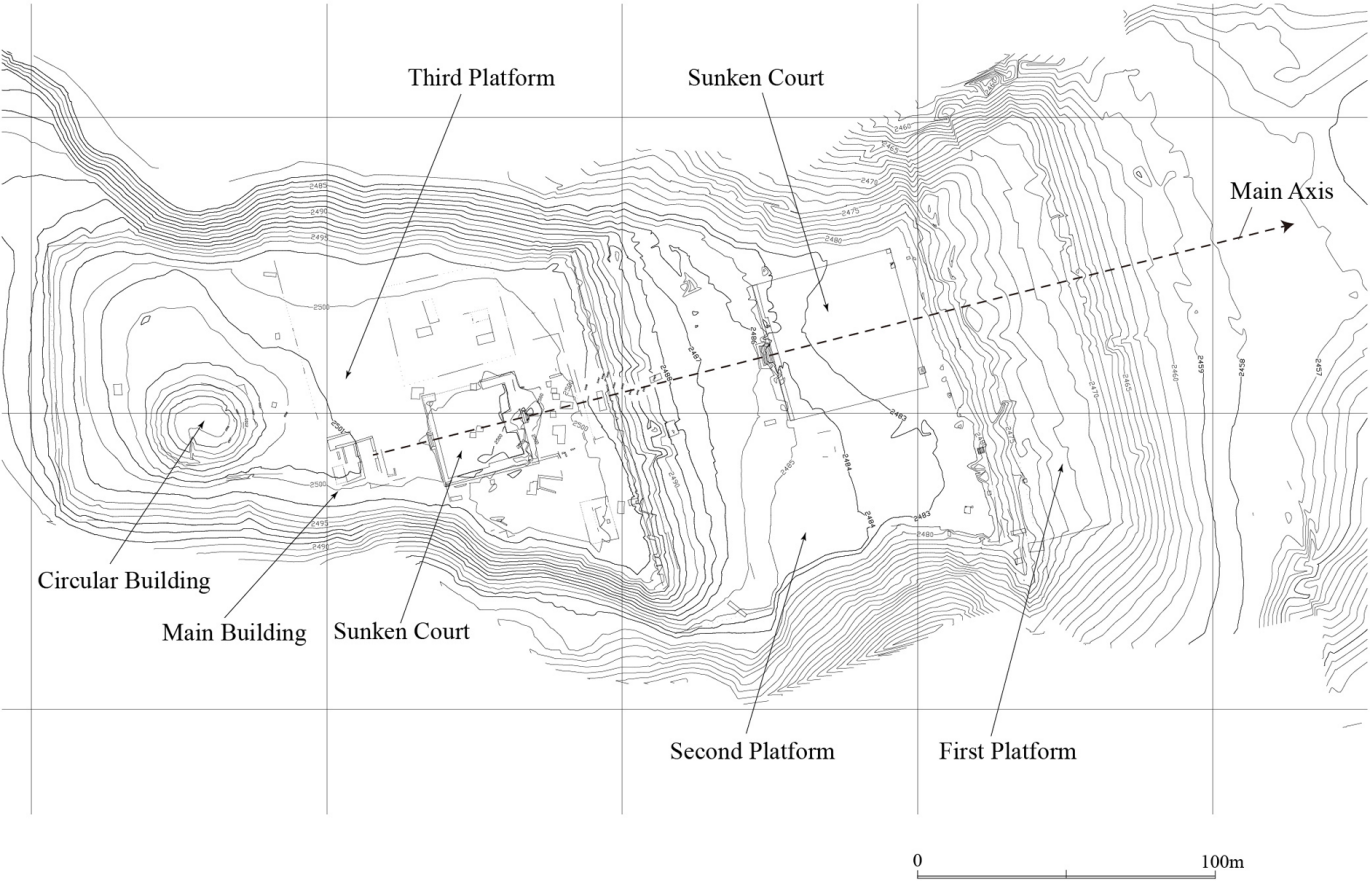
Map of the ceremonial complex at Pacopampa.

We began our archaeological project at Pacopampa in 2005, and have obtained new findings on socioeconomic inequality during the Formative Period. During that period, a sunken plaza surrounded by three low platforms was constructed at Pacopampa; the westernmost one was called “the Main Platform” by the Pacopampa Archaeological Project (Fig 2). This may have been the most important religious space in the overall complex.

An unusual human skeleton from the beginning of the Pacopampa II cultural phase (800–500 BC) (see below) was recovered from the Main Platform: it had cinnabar and azurite spread on its skull and a pair of gold earplugs, a pair of gold earrings, and shell objects [30]. The bright red cinnabar was sourced from cinnabar ore mined in Huancavelica over 850 km to the south [31]. A detailed examination of the neurocranium revealed the presence of a fronto-occipital type of artificial cranial deformation, which had not been found among human remains with burial goods indicating a lower social class [15]. Subsequent excavations yielded one perinatal individual in the Pacopampa I cultural phase (1200–800 BC) and eight adult individuals from the Pacopampa II cultural phase (800–500 BC), who belonged to an elite class. The precious goods found in their graves and their special burial treatment demonstrated that social inequality at Pacopampa may date back to at least the beginning of the Pacopampa II cultural phase. It is reasonable to assume that the precious objects, pigments, and artificial cranial deformations found can be considered proxies of social class there.

## Materials and methods

### Skeletal sample

Eleven field seasons from 2005 to 2015 yielded the remains of 104 individuals, comprising 38 non-adults and 66 adults (S1 Table). Archaeological artifacts, architecture, stratigraphy, and radiocarbon dating revealed that the chronological ages of the human remains belonged to two phases:

1. the Middle Formative Period (Pacopampa I, 1200–800 BC); and
2. the Late Formative Period (Pacopampa II, 800–500 BC).

Beta Analytic, a laboratory in Miami, Florida, conducted the radiocarbon dating of the bones.

The materials are curated by the Pacopampa Archaeological Project and temporally housed at the Center for Pacopampa Archaeological Project (Jr. Bolognesi, Centro Poblado de Pacopampa, Distrito de Querocoto, Provincia de Chota, Region Cajamarca, Peru) under the permissions of the Peruvian Ministry of Culture. They are accessible by others who get in contact with the Project. All necessary permits were obtained for the described study, which complied with all relevant regulations: the permissions of the National Institute of Culture of Peru from 2005 to 2010 (Permission Nos: 1108/INC, 1868/INC, 979/INC, 1061/INC, 815/INC, and 1403/INC) and those of the Peruvian Ministry of Culture from 2011 to 2015 (Permission Nos: 265-2011-DGPC-VMPCIC/MC, 593-2012-DGPC-VMPCIC/MC, 006-2013-DGPA-VMPCIC/MC, 363-2014-DGPA-VMPCIC/MC, and 270-2015-DGPA-VMPCIC/MC). No aspect of the materials or methods of this study needed to be approved by our institutions’ ethical committees. This study was conducted according to the Vermillion Accord on Human Remains (approved by the World Archaeological Congress at 1989)

### Age and sex estimation

We determined the ages of non-adult skeletons based on dental development, such as the formation of all crowns and roots and the eruption of each tooth [32]. For individuals whose ages we could not estimate by dentition, we measured their temporal and occipital bones [33, 34], ilia [32], and limb bones [35], and observed the degree of development and the closure of the occipital synchondrosis [36] as well as the extent of ossification and epiphyseal union of the pelvis and long bones [37]. We classified the ages in years of adults who were 15 years or older at the time of death into three categories: young (15–34 years); middle (35–54 years); and elderly (≥55 years), based on the chronological metamorphosis of the auricular surface of the ilium [38, 39], pubic symphysis [40–43], first ribs [44], and dental wear [45]. We determined the sex of individuals aged ≥15 based on a macroscopic assessment of pelvic and cranial traits [46–48]. Skeletons buried with gold and silver ornaments, pigments, and artificial cranial deformations were classified as elites, while those lacking such elements were deemed commoners.

### Examination criteria for trauma

We examined trauma macroscopically alone and recorded patterns based on the age at death, sex, social class, and chronological phase. Radiographic analyses were not available because the obtained permissions do not allow us to transport skeletal remains to our institutions for the analyses. The locations, types, and mechanisms of the trauma, and its healing processes were all considered. Fractures were categorized into transverse, oblique, spiral, depressed, crushed, wedge, greenstick, pathological, and stress fractures [49] and the skull fractures were further classified into blunt force fracture and sharp force fractures [50]. In terms of the mechanisms, this study classified fractures into direct and indirect types: the former are fractures produced by the direct application of force, and the latter are fractures occurring at a distance from the loading area [51]. In general, indirect and torsional forces cause oblique and spiral fractures and direct force causes transverse and depressed skull fracture [50, 51]. We also recorded the presence of complications of the fracture, such as delayed healing, pseudoarthrosis, poor alignment, bone shortening, osteomyelitis, or avascular necrosis of bone [49, 52].

The healing processes of fractures in living humans can be divided into six stages: hematoma formation, hematoma organization, fibrous callus formation, primary bony callus formation, secondary callus formation, and remodeling of callus [53]. Another and more concise classification separates healing into three stages: the inflammatory phase, lasting for 72 hours; the reparative phase, lasting about two weeks; and the remodeling phase, which begins in the middle of the reparative phase and may last for up to several years [49]. However, it is impossible to classify fracture in archaeological skeletons fully into these categories; this study classified the fractures to the degree possible, by whether they had reached the remodeling phase or not, according to the latter classification [49]. The remodeling phase is characterized by the remodeling of callus and smoothed fractured surface [49].

We identified perimortem trauma by the absence of new bone formations and the freshness of broken surfaces. We also used differences in stains and colors to distinguish perimortem trauma and taphonomic changes. We excluded fractures caused by morbidity, such as osteoporosis. Analyses of wound type, lethality, location on the body, demographic structure, burial places, burial goods, and settlement patterns were used to distinguish between accidental and violent injuries [2].

The observed sample used in this study is the individuals who had the adequate conservation of bones, especially crania, in order for lesions to be macroscopically visible. The prevalence of lesions was calculated by dividing lesions over these individuals. We used Fisher’s exact test to compare trauma frequencies among groups.

## Results

Of the 104 individuals, seven exhibited signs of trauma (6.7%), comprising fractures in the crania, facial bones, and limb bones, and dislocation in the elbow joint. The results of observation are summarized in S2 Table. There were no observation of fractures to the hand and foot bones and ribs.

All seven individuals with trauma were recovered from the third platform (Fig 2). One individual from the Middle Formative Period was buried at an access aisle connecting ritual areas, while six individuals from the Late Formative Period were buried within the temple in the northern area of the third platform.

### Depressed skull fractures

**Fig 3:**
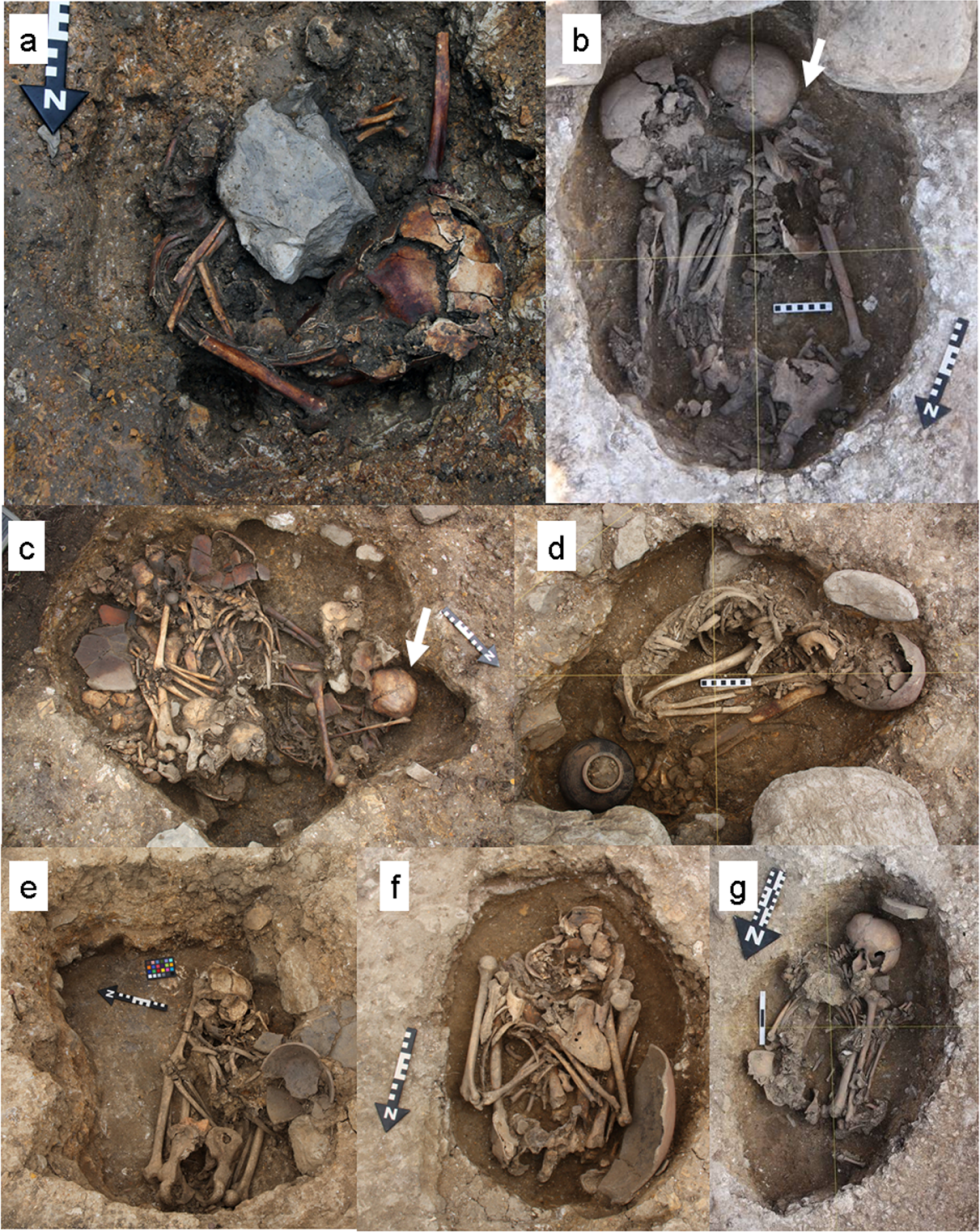
Burials of the individuals with trauma. (a) An adult female with depressed fractures in the right and left parietal bones (Specimen No. 11PC-C-Ent 1-H1). (b) A ≥55-year-old female with a depressed fracture in the left parietal bone (Specimen No. 12PC-B2-Ent 531-H2). (c) A 35–54-year-old male with depressed fractures in the frontal bone and the right parietal bone (Specimen No. 14PC-B2-Ent 537-H1). (d) A ≥55-year-old male with fractures in the right nasal bone and the left zygomatic bone (Specimen No. 14PC-A-Ent 7-H1). (e) A 15–34-year-old male with a fracture in the left fibula (Specimen No. 11PC-B2-Ent 516-H1). (f) A 15–34-year-old male with fractures in the left tibia and fibula (Specimen No. 12PC-B2-Ent 534). (g) A 35–54-year-old female with a dislocation in the right elbow joint (Specimen No. 13PC-B2-Ent 504-H2). Bold arrows show the trauma.

**Fig 4:**
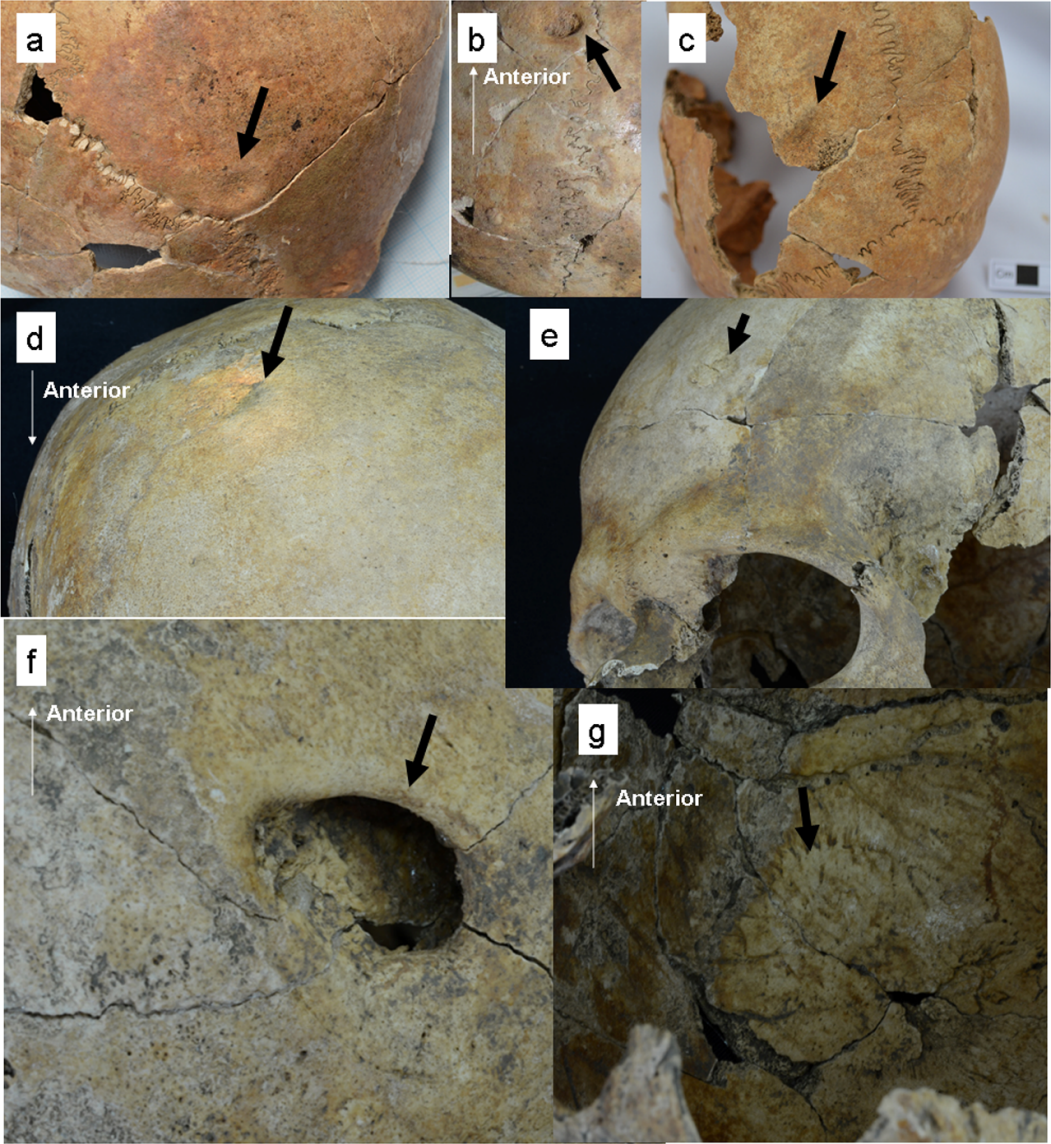
Depressed skull fractures. (a–b) Multiple fractures in the right and left parietal bones in an adult female (Specimen No. 11PC-C-Ent 1-H1). (c) A fracture in the left parietal bone in a ≥55-year-old female (Specimen No. 12PC-B2-Ent 531-H2). (d–g) Multiple fractures in the frontal bone and the right parietal bone in a 35–54-year-old male (Specimen No. 14PC-B2-Ent 537-H1). Bold arrows show the trauma.

We observed depressed skull fractures in individuals from both of the abovementioned chronological phases. An adult female (Specimen No. 11PC-C-Ent 1-H1), buried in an almost articulated position with a slingstone, was the earliest example found (Fig 3A). This individual was dated to 2950±30 ^14^C yr BP, in the Middle Formative Period. The depressed fracture is in the posterior part of the right parietal bone (Fig 4A); its shape is circular, with a diameter of almost 1 cm, and its depth is limited to the outer table. The fracture was classified as a blunt force fracture produced by the direct application of force. Another possible fracture in this individual is a circular depression with a diameter of almost 1 cm on the anterior part of the left parietal bone (Fig 4B); this fracture was also classified as a blunt force fracture, produced by the direct application of force to the cranial vault. The depressed area is surrounded by a smooth surface, while the center of the depression exposed a trabecular bone possibly caused by bone erosion due to post-fracture avascular necrosis. When damaged bone is cut off from its blood supply, the bone will die and the necrotic bone will finally be resorbed.

In both cases, the presence of a healing reaction with the formation of smooth edges around the fractures shows that they are antemortem fractures and that the individual survived after receiving the fractures. The healing process was classified into the remodeling phase.

For the Late Formative Period, we found depressed skull fractures in both sexes. The first such case was a ≥55-year-old female, buried in an articulated position with chrysocolla ornaments and pottery (Specimen No. 12PC-B2-Ent 531-H2) (Fig 3B). This individual was buried with a 35–54-year-old male (Specimen No. 12PC-B2-Ent 531-H1), which showed no trauma. The female individual exhibited a depression limited to the outer table on the posterior part of the left parietal bone (Fig 4C). The shape of the depressed fracture is circular, with a diameter of 20mm. The fracture was classified as a blunt force fracture produced by the direct application of force to the cranial vault. A healing reaction with the formation of smooth edge around the fracture suggests that it is an antemortem fracture and that the individual survived after suffering the fracture. The healing process was classified into the remodeling phase.

The second case involves multiple depressed fractures in a 35–54-year-old male (Specimen No. 14PC-B2-Ent 537-H1), who was buried in an articulated position with a chrysocolla ornament, pottery, fragments of a clay figure, and a copper product (Fig 3C). The individual was buried with a ≥55-year-old female (Specimen No. 14PC-B2-Ent 537-H2) and a 35–54-year-old female (Specimen No. 14PC-B2-Ent 537-H3), both of which showed no trauma. The individual exhibited three depressed skull fractures: in the right and left sides of the frontal bone and in the right parietal bone. These fractures were classified as blunt force fractures produced by the direct application of force to the cranial vault. The fracture on the right side of the frontal bone is almost circular, at 9.2 mm long and 9.9 mm wide (Fig 4D); it is shallow and limited to the outer table. The depression with smooth edges was linked with new bone formation. The fracture in the left side of the frontal bone displayed signs of healing; it was possibly caused by a depressed skull fracture (Fig 4E), although it did not show a clear depression, but rather formed new bone at 10.7 mm long and 8.9 mm wide. The most severe depressed fracture in the right parietal bone is elliptical in shape, 12.4 mm long and 20.8 mm wide (Fig 4F). In it, depressed debris, which adheres in part to the vault, entered the cranial cavity and may have compressed the brain substance. The internal table revealed striations radiating from the depression, which constitute evidence of new bone formation (Fig 4G). It is unknown whether the fractures were caused by a single episode, but the presence of multiple fractures implies intentional blows (rather than trauma due to an accident), and new bone formation with smooth edges around the fractures indicates that they are antemortem fractures and that the individual may have survived after receiving them. The healing process was classified into the remodeling phase.

### Facial bone fractures

**Fig 5:**
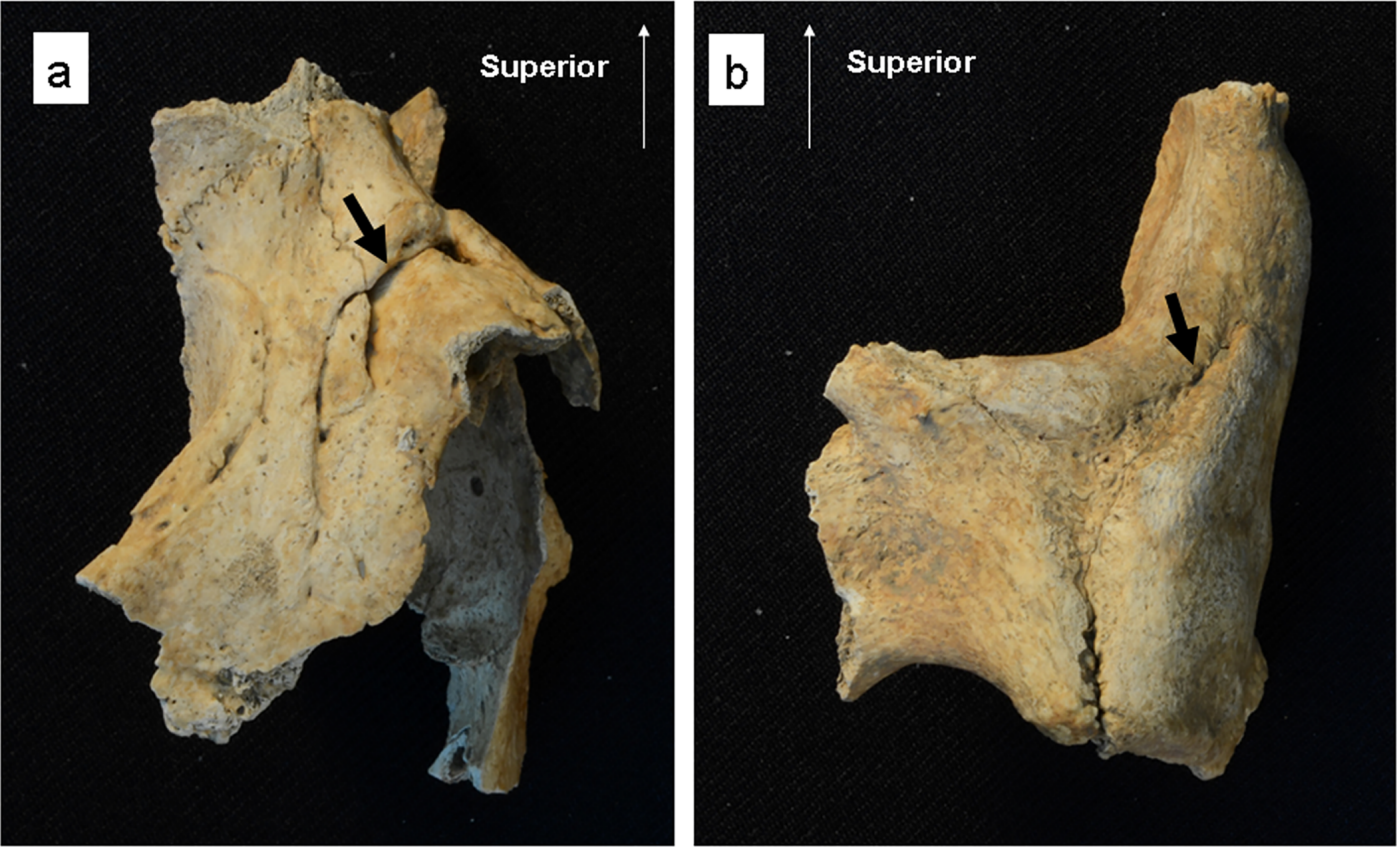
Facial bone fractures. Multiple fractures in the right nasal bone (a) and in the left zygomatic bone (b) in a ≥55-year-old male (Specimen No. 14PC-A-Ent 7-H1). Bold arrows show the trauma.

We only observed facial bone fractures in a ≥55-year-old male (Specimen No. 14PC-A-Ent 7-H1) from the Late Formative Period. This individual was buried in an articulated position with pottery (Fig 3D) and exhibited two fractures in the right nasal bone and left zygomatic bone respectively. The fractures were classified as a blunt force fracture produced by the direct application of force to the face. The former is a depression in the lower half of the right nasal bone, which was again fused with the upper part of the nasal bone after dislocation (Fig 5A). The dislocated right nasal bone moved the left nasal bone forward and then caused the left nasal bone to detach from the frontal process of the left maxilla. The latter fracture was in the left zygomatic bone, whose medial half was depressed compared to the lateral half (Fig 5B). While it is unknown whether the fractures resulted from a single episode, the presence of multiple areas of trauma implies intentional blows, rather than trauma due to an accident; in addition, new bone formation points to the possibility that they are antemortem fractures and the individual survived after being wounded. The healing process of these fractures was classified into the remodeling phase.

### Limb bone fractures and dislocation in the elbow joint

Limb bone trauma in Pacopampa specimens was found in healed fractures in two males and a dislocation in one female, all of which date to the Late Formative Period.

**Fig 6:**
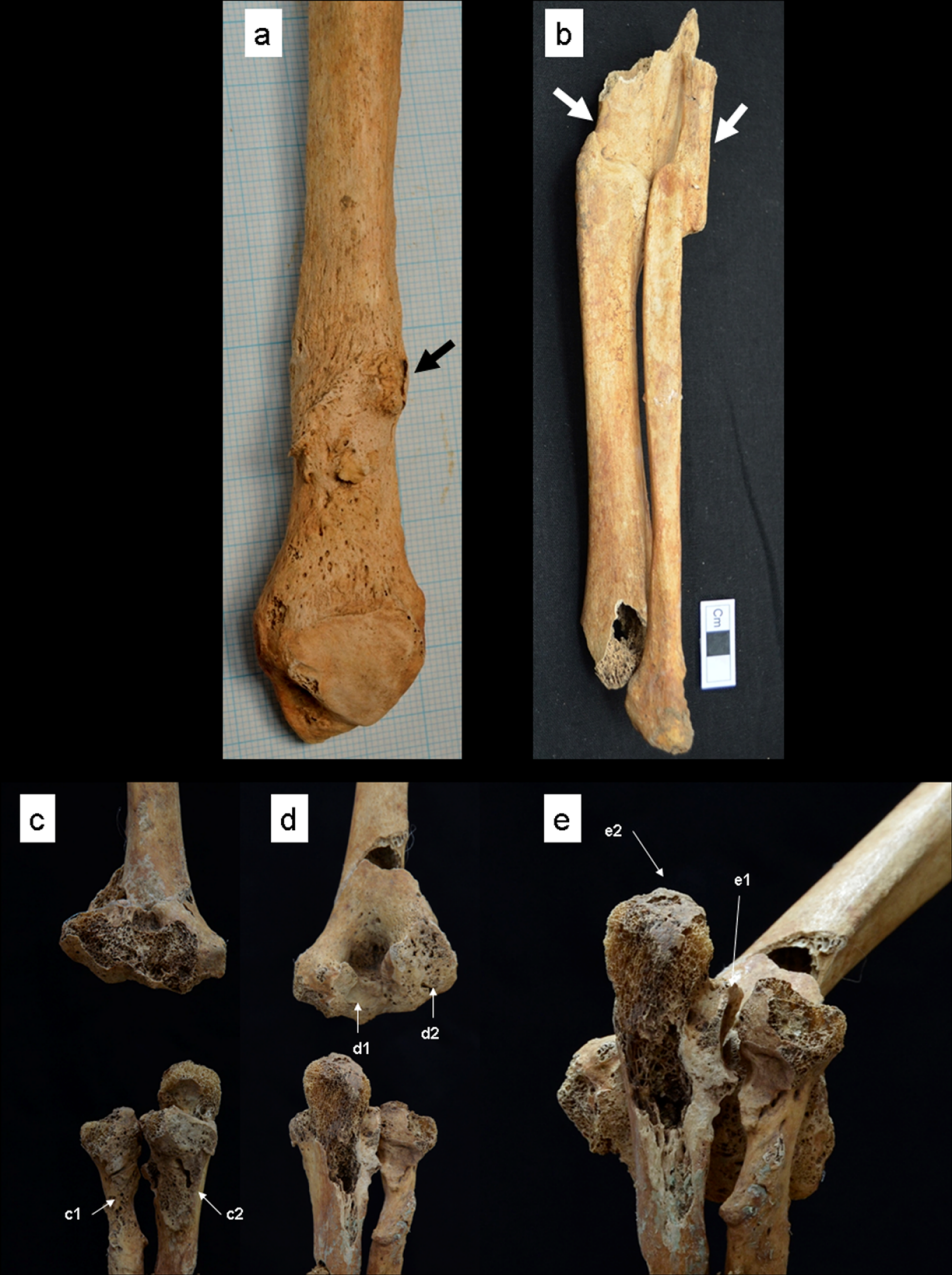
Limb bone fractures and dislocation in the elbow joint. (a) A fracture in the left fibula in a 15–34-year-old male (Specimen No. 11PC-B2-Ent 516-H1). (b) Fractures in the left tibia and fibula in a 15–34-year-old male (Specimen No. 12PC-B2-Ent 534). (c–e) A dislocation in the right elbow joint in a 35–54-year-old female (Specimen No. 13PC-B2-Ent 504-H2). Bold arrows show the trauma.

The first case of healed fracture is a limb bone fracture in a 15–34-year-old male (Specimen No. 11PC-B2-Ent 516-H1) who was buried in an articulated position with pottery (Fig 3E). The individual was dated to 2310±30 ^14^C yr BP. This case exhibited the distal end of the left fibula being moved in the anterior-lateral direction after the individual received the fracture (Fig 6A). Nutrient vascular foramina were detected in the fractured part, which is indicative of new bone formation during the healing process. This fracture was not associated with the tibial fracture. The fracture was possibly a transverse fracture produced by the direct application of force at a right angle to the bone, because the break of fracture seems to be horizontal. The presence of new bone formation at the fractured part indicates that it is an antemortem fracture and that the individual survived after being wounded. The healing process of the fracture was classified into the remodeling phase.

The second case involved a 15–34-year-old male (Specimen No. 12PC-B2-Ent 534) who was buried in an articulated position with pottery (Fig 3F). These fractures are severe, involving the left tibia and fibula, both of which were fractured midshaft (Fig 6B). The distal parts of the fractures had completely detached from the medial ones, moved in the anterior direction, and then fused with the medial parts again, keeping the distal and medial parts of the fractures dislocated. The bones were not fused together. Nutrient vascular foramina were detected in the fractured parts, which is indicative of new bone formation during the healing process. The positions of the fracture, dislocation, and fusion were identical between both bones when articulated; therefore, the fracture and healing of both bones must have occurred simultaneously. The fractures were classified as transverse fractures produced by the direct application of force at a right angle to the bones, because the break of fractures seems to be horizontal. The presence of new bone formation at the fractured parts indicates that they are antemortem fractures and that the individual survived after being wounded. The healing process of the fracture was classified into the remodeling phase. The lack of pseudarthrosis suggests the stabilization of fractured segments in both bones.

The only case of dislocation was that of the right elbow joint of a 35–54-year-old female (Specimen No. 13PC-B2-Ent 504-H2) who was buried in an articulated position with shell beads (Fig 3G). The individual exhibited severe deformities in the right humerus, the right radius, and the right ulna (Fig 6C–6E). The posterior surface of the lateral epicondyle in the right humerus formed a porous surface (similar to an articular surface) with osteophytes (d2 of Fig 6D), and the trochlea and olecranon fossa in the right humerus exhibited an irregular outline surrounded by lippings (d1 of Fig 6D). The right radius developed a porous depression similar to an articular surface in the lateral surface of the radial tuberosity (c1 of Fig 6C), which was articulated with the surface of the lateral epicondyle in the right humerus in the position of pronation (d2 of Fig 6D). The right ulna developed a porous depression similar to an articular surface in the ulnar tuberosity (27.0 mm long and 16.0 mm wide) (c2 of Fig 6C), which was articulated with the trochlea of the right humerus (d1 of Fig 6D). When the depression of the tuberosity of the ulna was articulated with the trochlea of the humerus, the coronoid process was located in the olecranon fossa (e1 of Fig 6E), and the olecranon was isolated from the original position of articulation, that is, the olecranon fossa (e2 of Fig 6E). The deformation was caused when the individual was subjected to high mechanical pressure from below in the position of pronation. The presence of osteophytes at the injured parts indicates that they are antemortem and that the individual survived after being wounded. It is not clear that the dislocation was associated with the Monteggia fracture (fracture of ulna with dislocation of radius), because the proximal half of the ulna was not preserved.

All these individuals survived fractures and dislocation due to a healing reaction; thus, it is easy to suppose that their deformities may have restricted their mobility and led them to develop some type of disability, affecting their daily lives. Either intentional blows or accidental falls may have caused the fractures and dislocation that we observed in the skeletons’ extremities. If the transverse fractures caused by the direct application of force to the bones are included in the trauma, they represent intentional violence.

### Trauma frequencies at Pacopampa

Trauma at Pacopampa fluctuated based on age at death, sex, social class, and chronological phase (S3 Table). Trauma was found in 0.0% (0/38) of non-adults, 10.6% (7/66) of adults, and 6.7% (7/104) of all specimens. If we exclude individuals of unknown sex, the trauma frequencies are 16.7% (4/24) in males, 10.3% (3/30) in females, and 13.0% (7/54) for both sexes.

Judging from the absence of luxury burial goods, pigments, and artificial cranial deformations, all seven individuals with trauma were commoners. The nine individuals identified as elite individuals and belonging to the Late Formative Period did not exhibit any signs of trauma (0.0%), while 20.0% (4/20) of male commoners and 12.0% (3/25) of female commoners—equating to 15.6% (7/45) for both sexes—did show signs of trauma. Although it seems that commoners of both sexes suffered more trauma than elite individuals, there was no significant difference between social classes (p = 0.59).

We detected trauma in two chronological phases. In the Middle Formative Period, we found a depressed skull fracture in one female in a group comprising two males and one female: thus, for the Middle Formative Period, trauma frequency was 100% (1/1) for females and 33.3% (1/3) for both sexes. Of the 22 males and 29 females from the Late Formative Period, the trauma detected in four males was composed of depressed skull fractures, facial bone fractures and lower limb bone fractures, while two females displayed a depressed skull fracture and a dislocation of the elbow joint respectively. For the Late Formative Period, therefore, trauma frequency was 18.2% (4/22) for males, 6.9% (2/29) for females, and 11.8% (6/51) for both sexes. If we exclude the nine elite individuals, the trauma frequency for commoners was 22.2% (4/18) for males, 8.3% (2/24) for females, and 14.3% (6/42) for both sexes. There was no clear change in trauma frequencies in either sex between the two chronological phases, whether the elite individuals were included (p = 0.35) or not (p = 0.41).

Among the individuals from the Late Formative Period, young adults exhibited less head trauma than middle-aged and older adults. Trauma frequency increased with age, from 4.8% (1/21) for those aged 15–34, to 10.0% (2/20) for those aged 35–54, up to 28.6% (2/7) for those 55 and older, though there was again no significant difference among them (p = 0.16). If we exclude elite individuals, trauma frequency was 7.1% (1/14) for individuals between 15 and 34, rising to 10.5% (2/19) for those aged 35 to 54 and 33.3% (2/6) for those 55 and older, showing the same age-related change, again non-significant (p = 0.27). We suspect that this is because older adults were exposed to more risk of trauma during their longer lifetimes. All individuals with trauma showed signs of healing rather than immediate death.

Trauma frequencies were 0.0% (0/2) for males and 100.0% (1/1) for females in the Middle Formative Period, 18.2% (4/22) for males and 6.9% (2/29) for females in the Late Formative Period, and 16.7% (4/24) for males and 10.0% (3/30) for females overall, though there were no significant differences between the sexes in the Middle Formative Period (p = 0.33), the Late Formative Period (p = 0.38), or overall (p = 0.69). In terms of specific types of trauma, we observed mild skull fractures and a dislocation of the elbow joint in females, and noted severe skull and limb bone fractures in males. Trauma in males became more severe compared to that of females as the chronological phases progressed. Indeed, we detected trauma only in females from the Middle Formative Period, while, in the Late Formative Period, it seemed that more males experienced trauma. Thus, the burden of trauma was not equally distributed among males and females.

## Discussion

### Violence at Pacopampa

**Fig 7:**
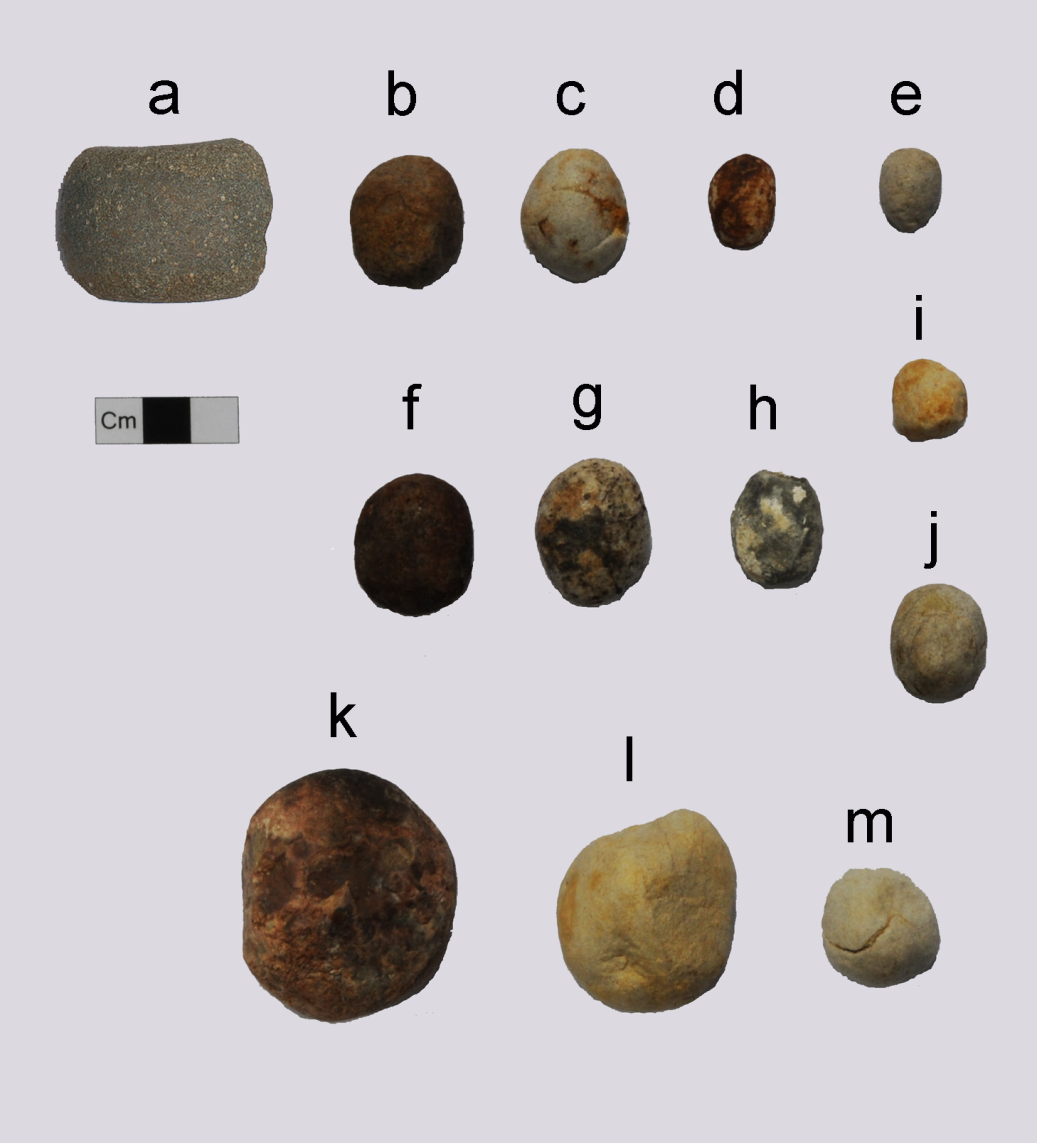
Stone tools. (a) Stone club head (Specimen No. 11PC-B-L881). (b–m) Slingstones (Specimen Nos. 09PC-C-L11, 12PC-B-L1158, 09PC-C-L387, 08PC-C-L48, 12PC-C-L974, 12PC-C-L1489, 10PC-C-N114, 10PC-C-N125, 08PC-C-L80, 09PC-C-L196, 12PC-B-L925, respectively).

The locations, types, and mechanisms of the trauma at Pacopampa indicate a predominance of direct fracture in skulls and limb bones. There were no observation of fractures to the hand and foot bones and ribs. The lack of fractures to them implies the biased distribution of fractures to the body. Cranial trauma accounted for 57.1% (4/7) of injuries, higher than for any other part of the body. It consisted of depressed skull fractures and facial bone fractures with healing. The multiple occurrences of trauma to the cranium and face suggest intentional and repeated blows to have been the cause, rather than accidents. Although the accidental injuries may have occurred during the daily life in the mountainous environment, it is safe to assume that these events would have led to higher probabilities of trauma on limb bones than crania. In general, depressed skull fractures and facial bone fractures are considered to be produced by intentional violent behaviors, while the limb bone fractures are the results of accidental falls, not by intentional blows with clubs, sling stones, or other weapons [2, 51, 52]. The round depression fractures were likely caused by close-proximity assaults with slingstones or thrown rocks, and broken noses may have resulted from armed combat or fistfights [2]. Stone tools used for weapons or ritual tools (for example, chipped stone points, stone club heads, and slingstones) (Fig 7A–7M) were excavated from the same strata, although they were not associated with the human remains. Iconography of Moche (AD 100–700) ceramics depicted these stone tools as weapons [54]. It is difficult to determine whether they were weapons, hunting equipment, or ritual tools, but we cannot rule out the possibility that they represented weapons.

The frequency of depressed skull fractures in adults at Pacopampa is 5.6% (3/54) in Pacopampa. There is no significant difference in frequencies of depressed fractures between males (1/24, 4.2%) and females (2/30, 6.7%) (p = 1), although the depth of the depression is more severe in the male victim than in the females. Comparing the frequencies of depressed skull fractures in adults among several archaeological sites, the frequency of Pacopampa is nearly equal to that of the Nasca culture (AD 1–750) (7/63, 11.1%) (p = 0.34) [55], but it is lower than the frequencies at Conchopata (7/27, 25.9%) (p = 0.01), Beringa (13/39, 33.3%) (p = 0.00), and La Real (32/102, 31.4%) (p = 0.00), where the Wari culture flourished (AD 650–800) [56]. The prevalence of lesions shown in this study and the comparative data was commonly calculated by dividing cranial lesions over the total crania. The presence of violent behavior at Pacopampa shows that people in the Formative Period did not lead such peaceful lives after all; however, it appears that the violence at Pacopampa was not intended to kill the opponents, as all the affected individuals exhibited signs of healing and experienced a lower degree of trauma than during the subsequent Wari culture. Death by violence did not represent the circumstances of the time.

The fact that commoners tend to exhibit more signs of trauma than elite individuals (though there is no significant difference between social classes, as mentioned above) runs contrary to the high risk of violence for the elites in subsequent eras among the Moche culture (AD 100–700), where elite males often engaged in ritual battles [56, 57]. At the site of La Real, elite males of the Wari culture also participated in ritual battles and had high frequencies of non-lethal head wounds with few to no parry fractures, while elite females had fewer injuries than males and are considered to have been protected from violence [56]. The trauma pattern detected in both sexes at Pacopampa is similar to that of females from La Real, and it is likely that Pacopampa elite males had not yet adopted a role as perpetrators of violence and were less exposed to the risk of trauma than commoners.

The previous studies’ interpretation of the reasons for violence in the Central Andes has depended on several pieces of evidence, such as the locations of skeletons’ trauma, healing reactions, association of the depressed skull fractures with other types of trauma, the demographic profile of the targeted population, burial goods, socioeconomic status, and settlement patterns [2, 23, 56, 58]. The full distribution pattern of trauma cannot be detected merely by looking at one side of the skull because we noticed trauma on both sides; however, cranial trauma was concentrated on the superior and posterior sides of the skull in females but on the anterior and superior sides of the skull in males. In general, the distribution pattern of trauma on the posterior half of the skull has been posited as attributable to raids or intra-household violence [56]. At Pacopampa, these explanations can be eliminated because the settlement pattern of the architecture is non-defensive, and most of the skeletons were buried in ritual places, not in living areas. In contrast, the distribution pattern of trauma on the anterior half of the skull indicates face-to face application of violence that may have been governed by strict rules, that is not to kill the person [59].

It is interesting that skull and facial bone fractures showed signs of healing and were not linked with parry fractures of the forearm. The traumatic pattern found at Pacopampa is similar to that found at La Real caused by a ritual battle called *tinku* [56, 59] or a ritual fight as a form of conflict resolution [56, 59], which are not necessarily exclusive. At La Real, fractures of upper limb bones were rare but there were cases of severe tibial fractures [59]. In *tinku*, combatants fought face-to-face or threw stones at each other; spilling blood during battle was regarded as an offering to the earth to pray for fertility [56, 59]. Men frequently participated in a ritual battle, *tinku*, but it occasionally involved females [56, 59]. Similarly, physical conflict resolution in the form of a ritualized club fight was performed mainly by men to resolve social tensions, following the ritualized rule that blows should be struck with wooden clubs to the head and shoulders [59]. These fights may not have caused fatal wounds and they were not associated with parry fractures of the forearm [59]. Tung [56, 59] cited a similar case among hunter-gatherers in California [60], reasoning that depressed fractures are not linked with parry fractures on the basis of the assumption that *tinku* or similar physical conflict resolution combatants did not guard against blows to the head. The similarity of the trauma patterns found at Pacopampa and La Real reflects shared experiences between the two archaeological settings. Although we do not advance an explanation regarding the cause of the lower limb bone fractures at Pacopampa, the common distribution patterns of trauma including severe cases of postcranial trauma between the two sites support this interpretation.

Warfare, raids, and intra-community violence leave traces on the skeletons that are difficult to discriminate from the consequences of ritual battle or physical conflict resolution. However, we conclude that violence in a ritual context is the most plausible explanation for the trauma at Pacopampa for the following reasons. First, at Pacopampa, all the trauma displayed evidence of healing, which suggests that the purpose of violence was to wound rather than kill an individual. This is contrary to the general trend in ritual sacrifice, which in the later eras was often associated with an act of killing reflective of a monopoly over life, death, and socioeconomic control through ritual [61]. Therefore, it is suggested that the elites’ role may not yet have been established in the nascent hierarchical society at Pacopampa and that violence in a ritual context may therefore not necessarily have produced the same results.

Second, the equal distribution of trauma between the sexes supports the idea that trauma was not caused by warfare but rather by rituals, as males were preferentially selected for warfare. It is true that more males received trauma in the later chronological phase, but on the whole, we observed an equal amount of trauma in both sexes.

Third, most of the individuals with trauma at Pacopampa were buried in the articulated position along with various burial goods in ritual places. This supports the idea that they were not enemies during conflicts but rather suffered injuries due to ritual violence that subsequently healed.

Fourth and most importantly, a lack of defensive settlement, fortification, and property destruction at Pacopampa, and the archaeological context of Pacopampa—a ceremonial complex—are consistent with this interpretation.

Although there is no proof of *tinku* or physical conflict resolution at Pacopampa, as no stone assemblages and few clubs for battle have been found, the lack of parry fractures of the forearm, together with the aforementioned observations, implies that trauma at Pacopampa may have been related to ritual practices and that a form of ritual battle to give the opponents the physical and psychological damage may have existed during the Formative Period. The presence of ritual violence in a society that built remarkably large ceremonial architecture and became socially stratified without any political control of surplus agricultural products shows that Pacopampa was home to a complex society, founded on ritual practices in a ceremonial center. This is early evidence of violence in a ritual context in Peru’s northern highlands.

### Possible cause of the emergence of violence at Pacopampa

Trauma was found in more than 10% of the skeletons in both chronological phases. Though there was no clear change in the trauma frequency, the severity of trauma increased as each chronological phase progressed: the individuals we examined experienced more severe fractures in the Late Formative Period than in the Middle Formative Period. It is generally accepted that population density, sedentism, social complexity, resource productivity, and environmental stability might explain the emergence of and changes in trauma [1]. However, in this case, the timing of trauma onset and changes did not coincide with the onset of sedentism and maize agriculture, which had occurred before the Formative Period [3, 4].

Civilization in the Central Andes developed in a unique way in that sedentism and agriculture had only a limited effect on social development. An alternative explanation for such a shift is circumscription theory [57], which posits that in places where resources were concentrated in limited places and inhabitants were subjected to circumscription (such as Peru’s coasts), population growth increased conflicts after exploitable areas became fully occupied, resulting in the creation of government and social stratification [62]. According to Torres-Rouff and Costa Junqueira [63], who incorporated circumscription theory into their research as the resource stress model used in their research, social disruptions and resource shortages led to a rise in conflict in the Late Intermediate Period (950–1400 AD) along the southern Andean coast. This is consistent with the hypothesis that resources and social stress influenced local populations on the southern coast [63].

When this theory is applied to Pacopampa, the construction and renovation of ceremonial architecture may have led people to gather there and create the circumscribed environment consistent with the model. The mountains surrounding Pacopampa are considered to have inevitably reached a critical limit for food production. Signs of violence are possible evidence of increasing social tensions caused by this circumscription. Given the limited data, the circumscription theory is a consistent plausible explanation of violence and social developments in the case of Pacopampa: if the aggregation of people caused circumscription and if social stratification resulted therefrom, then violence in a ritual context may have been a byproduct of the process of circumscription.

The stress of circumscription caused by resource degradation has been a preferred explanation for the violence all along the ancient Andean coast, but the application of this interpretation to Pacopampa is not straightforward. A zooarchaeological study indicated that deer hunting continued in the Final Formative Period, long after the introduction of camelids to the region, and that there was no sign of population reduction among the wild animals in the northern highlands [64]. The nutritional status of the human skeletal remains further suggests that a stable food supply sustained the Pacopampa community throughout the Formative period, at least in comparison with the coastal regions, because the prevalence of cribra orbitalia at Pacopampa is lower than in coastal populations [65]. Therefore, we need to inquire into the causes of violence through lines of evidence other than the substantial socioeconomic limitations, although we cannot completely rule out the circumscription theory.

At Pacopampa, where ritual activity flourished throughout the Formative Period, it has been suggested that rituals related to water and food production have been suggested based on the discovery of subterranean canal systems and shell ornaments intended for ritual use [30]. In the Late Formative Period, burials of individuals with trauma were concentrated in ritual places; therefore, ritual activity is considered to have become more important in the ceremonial center than before. Burials of individuals especially from the Late Formative Period showed various degrees of wealth, from burials of shamans with precious objects to the burials of individuals associated with a complete lack of goods. The presence of various kinds of burials in the same time period demonstrates the emergence of social inequality linked to the construction and renovation of ceremonial architecture and thriving rituals.

Furthermore, the severe but healed trauma concentrated on the cranium, and without defensive wounds that were found in several cases at Pacopampa appears to have been a result of fierce forces applied under controlled conditions. The controlled practice of violence seems to have an affinity with the cult of predatory animals, which delivered an account of violence through the display of fierce forces to the audience of the ritual practices. In the Central Andes, predators, especially feline, had been the key icon that reflected the religious outlook during the Formative Period [66–70]. In the temple of Chavín de Huántar, the portrayal of humans with fierce animal characteristics may have represented the incorporation of natural power in humans [71]. These anthropomorphized creatures emphasized the visible role of the high-ranking members of the cult and helped justified the elite’s monopoly over life, death, and socioeconomic control through ritual [71]. Furthermore, anthropomorphized creatures were also found on pottery, stone sculpture, and wall relief at Pacopampa; we can suspect that these figures may have exercised fierce forces on victims in ritual practices. If we apply this explanation, we see that violence in a ritual context may have contributed to the dominance over the people by an elite class. Violence may have become an element of ritual activity and the basis for social development, particularly where it was incorporated into rituals by taking on a new meaning of sacredness in ritual places. It seems likely that the severity and patterns of trauma that occurred morphed as each chronological phase progressed, and that its expression varied according to stratified social classes, which did not provide or coincide with an accurate reflection of sedentary lives and agriculture, but rather came along the appearance of social complexity.

## Supporting information

S1 Table Age at death and sex composition of pacopampa skeletal remains. (XLS)

S2 Table Trauma at Pacopampa. (XLS)

S3 Table Composition of males and females with Trauma. (XLS)
